# Assessment of glycohemoglobin, plasma glucose curve and C-reactive protein as complementary predictors to diagnose prediabetes: a transversal study

**DOI:** 10.1186/1758-5996-7-S1-A153

**Published:** 2015-11-11

**Authors:** Marcelo da Silva Biavaschi, Roberta Schmatz, Jessié Martins Gutierres, Pauline da Costa, Luciane Flores Jacobi, Thabara Campos, Isadora Scaburi, Maria Rosa Chitolina Schetinger, Vera Maria Melchiors Morsch

**Affiliations:** 1 UFSM, Santa Maria, Brazil

## Background

The shape of the plasma glucose curve has been considered as a predictor for diabetes in the future, and it could be used to diagnose prediabetes.

## Objective

This study aims to assess the relationship between glycohemoglobin, C-reactive protein (CRP), oxidative stress markers levels and the shape of the plasma glucose curve during the oral glucose tolerance test (OGTT).

## Material and methods

A transversal study was carried out with 59 non-diabetic subjects with increased diabetes risk who underwent an OGTT to analyze glycemia, insulin, glycohemoglobin, lipids, C-reactive protein, malondialdehyde and carbonyl protein. Glycemia was assessed at fasting, 30, 60 and 120 min.

## Results

Overall, 26 individuals had prediabetes, 28 did not have prediabetes (22 had 1h-OGTT>fasting glycemia, 6 had 1h-OGTT≤fasting glycemia) and 5 had diabetes. The 1h-OGTT≤fasting glycemia and diabetes groups were excluded, and then a non-parametric test (Mann-Whitney U test) was used to analyze data. There were no statistical differences between 1h-OGTT>fasting glycemia and prediabetes groups in the analysis of glycohemoglobin [5.15 (4.8-5.4); 5.20 (5.10-5.4); p=0.17 ], C-reactive protein [3.70 (1.8-8.9); 3.96 (1.45-7.13); p=0.38], lipids and malondialdehyde, but there were differences in fasting, 30, 60 and 120 min glycemia, insulin level [11(8-13); 13(10-18); p=0.04], Homeostatic Model Assessment-IR [2.45(1.93-2.95); 3.30(2.51-5.02); p=0.00] and carbonyl protein [0.48 (0.42-0.58); 0.57 (0.5-0.76); p=0.02].

## Conclusions

These results identify predictors to increased cardiovascular and diabetes risk in individuals without prediabetes and with 1h-OGTT>fasting glycemia, which are similar to predictors in individuals with prediabetes established by the American Diabetes Association criteria.

